# Identification of anti‐inflammatory vesicle‐like nanoparticles in honey

**DOI:** 10.1002/jev2.12069

**Published:** 2021-02-12

**Authors:** Xingyi Chen, Baolong Liu, Xingzhi Li, Thuy T. An, You Zhou, Gang Li, Judy Wu‐Smart, Sophie Alvarez, Michael J. Naldrett, James Eudy, Gregory Kubik, Richard A. Wilson, Stephen D. Kachman, Juan Cui, Jiujiu Yu

**Affiliations:** ^1^ Department of Nutrition and Health Sciences University of Nebraska‐Lincoln Lincoln Nebraska USA; ^2^ Department of Computer Science and Engineering University of Nebraska‐Lincoln Lincoln Nebraska USA; ^3^ Center for Biotechnology University of Nebraska‐Lincoln Lincoln Nebraska USA; ^4^ Department of Plant Pathology University of Nebraska‐Lincoln Lincoln Nebraska USA; ^5^ Department of Entomology University of Nebraska‐Lincoln Lincoln Nebraska USA; ^6^ Nebraska Center for Biotechnology, University of Nebraska‐Lincoln Proteomics and Metabolomics Facility Nebraska USA; ^7^ Department of Genetics Cell Biology and Anatomy University of Nebraska Medical Center, 985915 Nebraska Medical Center Omaha Nebraska USA; ^8^ Genomics Core Facility, University of Nebraska Medical Center Omaha Nebraska USA; ^9^ Department of Statistics University of Nebraska‐Lincoln Lincoln Nebraska USA

**Keywords:** extracellular vesicles, exosomes, honey, inflammation, nanoparticles, NLRP3 inflammasome, vesicles

## Abstract

Honey has been used as a nutrient, an ointment, and a medicine worldwide for many centuries. Modern research has demonstrated that honey has many medicinal properties, reflected in its anti‐microbial, anti‐oxidant, and anti‐inflammatory bioactivities. Honey is composed of sugars, water and a myriad of minor components, including minerals, vitamins, proteins and polyphenols. Here, we report a new bioactive component‒vesicle‐like nanoparticles‒in honey (H‐VLNs). These H‐VLNs are membrane‐bound nano‐scale particles that contain lipids, proteins and small‐sized RNAs. The presence of plant‐originated plasma transmembrane proteins and plasma membrane‐associated proteins suggests the potential vesicle‐like nature of these particles. H‐VLNs impede the formation and activation of the nucleotide‐binding domain and leucine‐rich repeat related (NLR) family, pyrin domain containing 3 (NLRP3) inflammasome, which is a crucial inflammatory signalling platform in the innate immune system. Intraperitoneal administration of H‐VLNs in mice alleviates inflammation and liver damage in the experimentally induced acute liver injury. miR‐4057 in H‐VLNs was identified in inhibiting NLRP3 inflammasome activation. Together, our studies have identified anti‐inflammatory VLNs as a new bioactive agent in honey.

## 1 INTRODUCTION

Honey, a naturally sweet product processed by honey bees from the nectar of flowers, has been used by humans worldwide as a nutrient, an ointment, and a medicine for many centuries (Bogdanov et al., 2008; Pasupuleti et al., 2017; Samarghandian et al., 2017). Honey is an excellent energy source because of its high sugar content. As an ointment, honey is effective in promoting healing of skin burns and wounds. Oral consumption of honey for medicinal purposes has been used since ancient times to treat throat infection, constipation, haemorrhoids, and other diseases. Modern scientific evidence suggests that honey helps in management of a variety of diseases, including cardiovascular disease, liver disease, and cancer, due to its anti‐inflammatory, anti‐microbial, and anti‐oxidant properties (Pasupuleti et al., 2017; Samarghandian et al., 2017).

Honey contains sugars, water, and a small portion of other components such as minerals, vitamins, and enzymes, as well as polyphenols (Pasupuleti et al., 2017), which are considered to be the major bioactive components in honey (Cianciosi et al., 2018). However, it is possible that honey contains other bioactive compounds that are yet to be discovered. Extracellular vesicles (EVs), or vesicle‐like nanoparticles (VLNs) have been recently identified in many commonly consumed foods, such as bovine milk, grapes, broccoli, carrots, ginger, and apples (Munir et al., 2020; Zempleni et al., 2018). Dietary EVs and VLNs are tiny membrane‐enclosed particles approximately 80–300 nm in diameter that contain biomolecules, including lipids, RNAs, and proteins (Chen et al., 2019; Mu et al., 2014). Bovine milk‐derived nanoparticles have been verified as EVs because EV‐specific markers, such as CD63, CD81, and Tumor susceptibility gene 101 (Tsg101), are found in these particles (Wolf et al., 2015). However, such vesicle‐specific markers have not been established in the nanoparticles extracted from vegetables and fruits; therefore, these particles are often termed exosome‐like nanoparticles, or vesicle‐like nanoparticles. Dietary EVs and VLNs have diverse beneficial effects on consumer health (Teng et al., 2018; Wu et al., 2019; Zhang et al., 2016). It has remained unresolved whether honey contains VLNs with any functions.

Dysregulated activation of the nucleotide‐binding domain and leucine‐rich repeat related (NLR) family, pyrin domain containing 3 (NLRP3) inflammasome is implicated in the aetiology of many disorders, including gout, Alzheimer's disease, and type 2 diabetes (Guo et al., 2015; Lamkanfi & Dixit, 2012). The NLRP3 inflammasome is an inflammatory signalling platform composed of the sensor NLRP3, the adaptor apoptotic speck protein containing a caspase recruitment domain (ASC), and the effector caspase‐1 (Casp1) (Lamkanfi & Dixit, 2012). Two distinct signals are required to activate the NLRP3 inflammasome (Guo et al., 2015; He et al., 2016). A priming signal, such as lipopolysaccharide (LPS), induces expression of the *Nlrp3* and *Il1b* genes. A second activating stimulus, which can be cholesterol crystals, microbial toxins, or extracellular ATP, triggers assembly of the inflammasome platform. After the inflammasome complex is formed, Casp1 cleaves itself to generate the active Casp1 p10 and p20, which process the precursors pro‐interleukin (IL)‐1β and pro‐IL‐18 to produce potent pro‐inflammatory cytokines IL‐1β and IL‐18. Activation of the NLRP3 inflammasome also causes pyroptotic cell death and therefore releases damage‐associated molecular patterns (DAMPs) to amplify inflammatory responses (Guo et al., 2015; He et al., 2016). Recently, honey has been found to ameliorate chronic inflammation and hepatic injury in rats challenged with a high‐fat diet (HFD), and such protective functions of honey are at least partially mediated through targeting the NLRP3 inflammasome (Xiao et al., 2016). However, it is not clear whether honey directly inhibits activation of the NLRP3 inflammasome. In the current study, we investigate whether honey contains VLNs and, if so, whether such VLNs have any influence on NLRP3 inflammasome activity.

## 2 RESULTS

### 2.1 Honey contains VLNs

**FIGURE 1:**
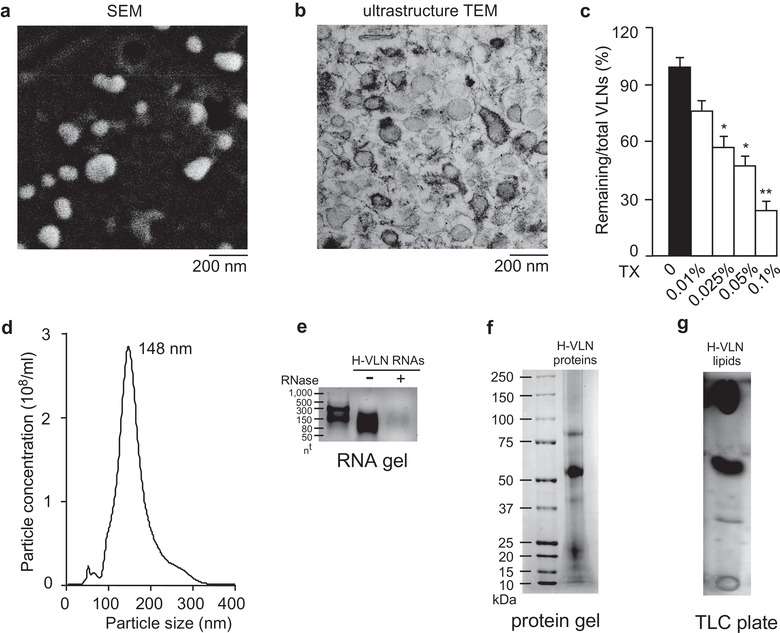
Honey contained VLNs. [a. Representative SEM image of H‐VLNs. b. Representative ultrastructure TEM image of H‐VLNs. c. Triton X‐100 (TX) lysis of H‐VLNs. Different concentrations of TX were incubated with H‐VLNs followed by vigorous agitation. d. Size of H‐VLNs measured using a NanoSight NS300 instrument. e. RNA gel of H‐VLN RNAs. RNAs were incubated with (indicated by ‘+’) or without (indicated by ‘‐’) RNase (10 μg/ml) at 37°C for 30 min before they were loaded on a 2.5% agarose gel. nt: nucleotides. f. Protein gel of H‐VLN proteins. Proteins were run on a 4%–12% Bis‐Tris protein gel and visualized with Coomassie blue. g. TLC analysis of H‐VLN lipids. A TLC silica gel plate was used to run the lipids. Data were presented as mean ± SEM. N = 3. *P < 0.05, **P < 0.01 relative to H‐VLNs without detergent treatment (black bar)].

Monofloral unprocessed manuka honey was diluted in cold phosphate‐buffered saline (PBS) and subjected to VLN extraction (Chen et al., 2019). A total of 7.5 ± 1.6 × 10^10^/g of VLNs were obtained from honey (Table S1). When these honey‐derived VLNs (H‐VLNs) were examined under scanning electron microscopy (SEM), they were shown to be individual tiny ball‐like spheres with diameters ranging from 120 nm to 180 nm (Figure 1a). Under ultrastructure transmission electron microscopy (TEM), these H‐VLNs appeared to have a membrane‐enclosed vesicle‐like structure (Figure 1b). Triton X‐100, a non‐ionic detergent that interrupts lipid‐protein and lipid‐lipid interactions and thus compromises the membrane integrity, is often used to distinguish membrane‐bound particles from protein aggregates (Gyorgy et al., 2011; Osteikoetxea et al., 2015). Triton X‐100 incubation followed by vigorous agitation using a vortex mixer effectively lysed and therefore reduced the number of H‐VLNs (Figure 1c), suggesting that H‐VLNs were mainly membrane‐bound particles. Nanoparticle tracking analysis (NTA) indicated that the majority of H‐VLNs were approximately 148 nm in diameter (Figure 1d). Purified naked RNAs from H‐VLNs were largely small‐sized RNAs and sensitive to degradation by RNase (Figure 1e). A series of protein bands were found when the proteins of H‐VLNs were separated on a protein gel and visualized using Coomassie staining (Figure 1f). Thin‐layer chromatography (TLC) analysis showed distinct lipid species in H‐VLNs (Figure 1g). The concentrations of RNAs, proteins, and lipids in H‐VLNs were approximately 31 ng/10^10^ particles, 9 μg/10^10^  particles, and 216 μg /10^10^  particles, respectively (Table S2).

The proteins in H‐VLNs were analyzed using liquid chromatography‐mass spectrometry (LC‐MS/MS). Because honey consists of the nectar harvested from the flowers and repeatedly regurgitated by honey bees, thus possibly containing proteins from both honey bees and plants (Bogdanov et al., 2008), the data were searched using the reference proteome database for both *Apis mellifera* and *viridiplantae*. A total of 142 plant‐derived proteins and 82 honey bee‐derived proteins were identified in H‐VLNs (Table S3). Many honey bee‐derived proteins were uncharacterized, therefore hindering further evaluation and analysis. Interestingly, a number of plasma transmembrane proteins from plants, such as plasma membrane ATPase, sugar transport protein 14‐like, cation H^+^ antiporter 19‐like protein, and major facilitator superfamily sugar transporter, were identified in H‐VLNs. In addition, Ras‐related protein and clathrin heavy chain, cytosolic proteins that are known to associate with plasma membrane (Magee & Marshall, 1999; Royle, 2006), were found in H‐VLNs. The presence of multiple plant‐derived plasma transmembrane proteins and plasma membrane‐associated cytosolic proteins in H‐VLNs further suggested vesicle‐like features of these particles.

To examine whether VLNs are generally present in honey, five more honeys, including one fresh polyfloral honey harvested directly from hives on the University of Nebraska‐Lincoln campus (UNL‐fresh) and four polyfloral honeys from local Nebraska farms (NE‐unprocessed, NE‐processed) or from mixed sources (Mixed‐unprocessed, Mixed‐processed) purchased at a local grocery shop, were subjected to VLN extraction. Unprocessed honey is honey that does not undergo any further process after harvest, whereas processed honey is lightly heated and filtered. All these honeys contained VLNs, although the particle yields ranged from 0.9 × 10^10^/g to 8.5 × 10^10^/g (Table S1). NTA showed that the sizes of VLNs from these five honeys varied from 142 nm to 156 nm in diameter (Figure  S1a). VLNs from three honeys (UNL‐fresh, NE‐unprocessed, and NE‐processed) were further verified to contain small‐sized RNAs (Figure  S1b), proteins (Figure  S1c), and lipids (Figure  S1d). Therefore, VLNs were found to be present in monofloral and polyfloral honeys from different sources, and these VLNs were composed of RNAs, proteins and lipids.

### 2.2 H‐VLNs suppressed activation of the NLRP3 inflammasome

**FIGURE 2:**
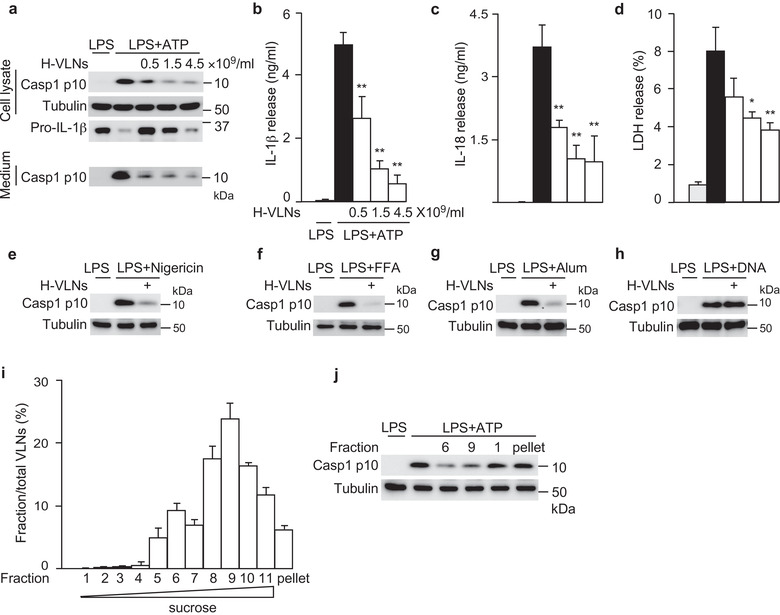
H‐VLNs specifically suppressed activation of the NLRP3 inflammasome. [a‐d. The primary macrophages were pretreated with H‐VLNs for 16 h, followed by addition of LPS + ATP to activate the NLRP3 inflammasome. H‐VLNs inhibited the level of Casp1 p10 in the cell lysate and culture medium (a), IL‐1β secretion (b), IL‐18 secretion (c), and pyroptosis (d). Lactate dehydrogenase (LDH) release assay was used to indicate the level of pyroptosis. e‐h. H‐VLNs inhibited Casp1 autocleavage when the NLRP3 inflammasome was activated with nigericin (e), free fatty acid (FFA) (f), or alum (g). H‐VLNs had marginal effects on Casp1 autocleavage upon AIM2 inflammasome activation (h). In e‐h, 4.5×10^9^/ml of H‐VLNs were used. i‐j. H‐VLNs were fractionated using sucrose gradient. Fractions were collected at 1 ml each and named as fractions 1‐11 from top to bottom. The particle number in each fraction was measured and presented as a percentage of the total particle numbers (i). The particles in fractions 6 and 9, the light particles in fraction 1, and the aggregates in pellet were collected and assessed for their inhibitory effects on NLRP3 inflammasome‐mediated Casp1 autocleavage (j). Data were presented as mean ± SEM. N = 3. *P < 0.05, **P < 0.01 relative to BMDMs treated with LPS + ATP (black bar). Tubulin showed equivalent loading of cell lysates].

Next, we assessed whether H‐VLNs had any influence on NLRP3 inflammasome activity. H‐VLNs from manuka honey were incubated with bone marrow‐derived macrophages (BMDMs), followed by activation of the NLRP3 inflammasome using the priming signal LPS and the activating signal ATP. Treatment with H‐VLNs in BMDMs inhibited all the downstream events of NLRP3 inflammasome activation, including generation of Casp1 autocleavage product Casp1 p10 (Figure 2a), secretion of cytokine IL‐1β and IL‐18 in the medium (Figure 2b and Figure 2c), and pyroptotic cell death (Figure 2d). In addition, H‐VLNs suppressed NLRP3 inflammasome activity when it was activated by different stimuli, including nigericin (Figure 2e), free fatty acid (Figure 2f), and alum (Figure 2g). Macrophages also contain a related inflammasome, Absent in Melanoma 2 (AIM2). In response to DNA in the cytoplasm in viral and bacterial infection, the sensor AIM2 recruits ASC and Casp1 to form the AIM2 inflammasome complex, which also leads to the autocleavage of Casp1 to generate Casp1 p10 and p20 (Guo et al., 2015; Lamkanfi & Dixit, 2012). However, when the AIM2 inflammasome was activated with LPS and DNA, H‐VLNs had marginal effects on Casp1 autocleavage (Figure 2h), suggesting that H‐VLNs specifically targeted the NLRP3 inflammasome.

To examine whether the vesicle‐like nanoparticles or other contaminants in H‐VLN preparation were responsible for anti‐NLRP3 inflammasome function, H‐VLNs were further fractionated using a sucrose gradient. The majority of H‐VLNs (92%) were distributed in the fraction 5–11, with a major peak at fraction 9 and a small peak at fraction 6 (Figure 2i). The particles from fraction 6 or 9 demonstrated potent anti‐NLRP3 inflammasome function (Figure 2j). Any light particles from the top layer (fraction 1) or aggregates in pellet were also collected, but none of them showed inhibitory effects on the NLRP3 inflammasome (Figure 2j). Together, these results suggested that the vesicle‐like nanoparticles in the H‐VLN preparation were responsible for inhibiting the NLRP3 inflammasome.

We noted that the protein level of pro‐IL‐1β in cell lysates decreased in a dose‐dependent manner when BMDMs were incubated with increasing amounts of H‐VLNs (Figure 2a). Expression of the *Il1b* gene was dramatically upregulated upon LPS treatment because LPS activates Nuclear factor‐κB (NF‐κB), a master transcriptional regulator that controls transcription of many inflammatory genes, including *Il1b, Il6*, and *Tnf* (Liu et al., 2017). To assess the effects of H‐VLNs on NF‐κB signalling, we first measured the levels of cytokine IL‐6 and tumor necrosis factor alpha (TNFα) in the culture medium. H‐VLNs reduced the level of IL‐6 in the medium but had marginal effects on TNFα secretion (Figures S2a and S2b). We further assessed the impact of H‐VLNs on transcription of inflammatory genes and found that H‐VLNs inhibited the expression of *Il6, Tnf*, and *Il1b* genes, although the inhibitory potencies varied — very strong inhibition on *Il6* gene and relatively modest inhibition on *Il1b* gene (Figure S2c‐S2e). Therefore, it seemed that H‐VLNs inhibited not only activation of the NLRP3 inflammasome but also NF‐κB signalling.

H‐VLNs from the other five honeys (UNL‐fresh, NE‐unprocessed, NE‐processed, Mixed‐unprocessed, and Mixed‐processed) were also tested for their anti‐inflammasome activity. The local honeys, including UNL‐fresh, NE‐unprocessed, and NE‐processed, consistently inhibited IL‐1β release and Casp1 autocleavage during NLRP3 inflammasome activation (Figure S3a‐S3e). The anti‐inflammasome effects of H‐VLNs from honey of mixed sources (Mixed‐unprocessed and Mixed‐processed) varied widely among three independent experiments and generally had less potent inhibitory effects on NLRP3 inflammasome activity (Figure  S3c, Figure  S3f, and Figure  S3g). H‐VLNs from these five honeys dramatically increased IL‐6 level (Figure S4a and Figure S4c) but showed no effects on TNFα level in the culture medium (Figure S4b and Figure S4d). Occasionally, we also noticed that H‐VLNs from certain batches of manuka honey showed the same effects as other honeys—they increased IL‐6 level in the medium. However, in general, H‐VLNs from manuka honey demonstrated consistent inhibition of both the NLRP3 inflammasome and IL‐6 secretion. Because manuka honey could be easily tracked by batch numbers and readily obtained from the local grocery shop in any season, it was selected for extraction of H‐VLNs for the following mechanistic and in vivo studies.

Other beehive products, including nectar, pollen, and royal jelly, were harvested on the UNL campus and subjected to VLN extraction. VLNs from nectar, which was freshly collected and minimally processed by the honey bees, inhibited NLRP3 inflammasome‐mediated Casp1 autocleavage (Figure S5a), suggesting that plant‐originated nanoparticles in the nectar may critically contribute to the anti‐inflammasome activity of H‐VLNs. However, VLNs from either plant‐originated pollen or bee‐secreted royal jelly had marginal influence on NLRP3 inflammasome activity (Figure S5b and Figure S5c).

### 2.3 H‐VLNs inhibited formation of the NLRP3 inflammasome platform

**FIGURE 3:**
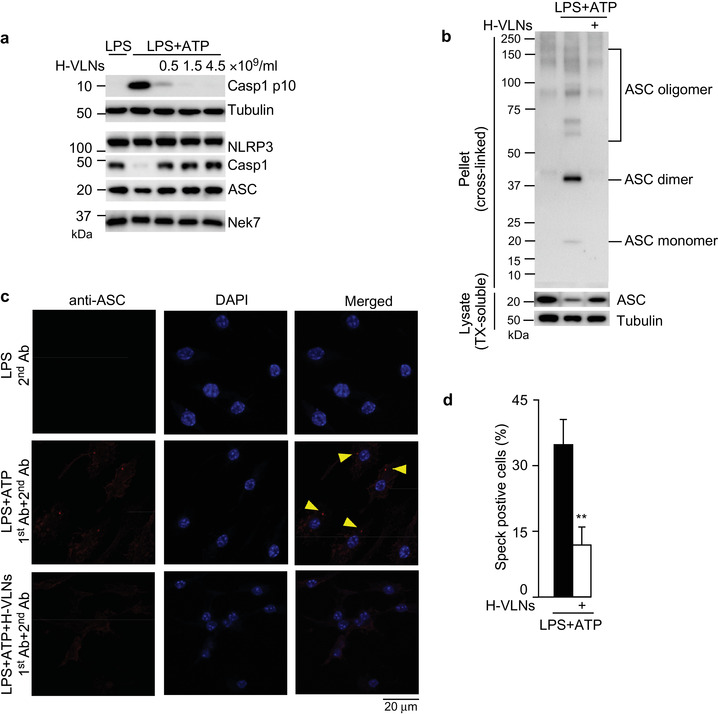
H‐VLNs inhibited formation of the NLRP3 inflammasome platform. [BMDMs were incubated with H‐VLNs for 16 h, treated with LPS + ATP to activate the NLRP3 inflammasome, and subjected to immunoblot analysis or immunofluorescence staining. a. H‐VLNs had marginal impact on the protein levels of NLRP3, Casp1, ASC, or Nek7. Tubulin showed equivalent loading of cell lysates. b. H‐VLNs blocked ASC oligomerization. TX: Triton X‐100. c. Representative immunofluorescence images of BMDMs. Casp1 inhibitor VX765 (10 μM) was added to the cells 30 min before ATP treatment to stabilize the NLRP3 inflammasome platform, which was visualized as a red speck. 1st Ab: anti‐ASC rabbit antibody; 2nd Ab: Alexa‐Fluor‐594‐conjugated anti‐rabbit antibody. 4’,6‐Diamidino‐2‐phenylindole (DAPI) was used to stain nuclei. d. Quantification of the inflammasome speck positive cells. Data were presented as mean ± SEM. N = 3. **P < 0.01 relative to BMDMs treated with LPS+ATP (black bar). In b‐d, 1.5 × 10^9^/ml of H‐VLNs were used].

The molecular mechanism by which H‐VLNs suppressed NLRP3 inflammasome activity was further investigated. First, the protein levels of inflammasome subunits were examined; H‐VLNs had no impact on the protein levels of NLRP3, ASC, and Casp1 (Figure 3a). The protein never in mitosis gene a (NIMA)‐related kinase 7 (NEK7), a critical mediator of NLRP3 inflammasome activation (He et al., 2016; Shi et al., 2015), has recently been shown to bridge adjacent NLRP3 subunits to facilitate NLRP3 oligomerization (Sharif et al., 2019). However, its level was not changed by H‐VLN treatment (Figure 3a). Oligomerized NLRP3 forms a platform to recruit ASC and induce the formation of ASC filaments, which subsequently recruit Casp1 and promote the polymerization of Casp1 (Lu et al., 2014). H‐VLNs blocked the formation of ASC oligomerization (Figure 3b). The assembled NLRP3 inflammasome is a multimeric protein complex with high molecular mass, which can be visualized as a speck through immunofluorescence staining using an anti‐ASC antibody (He et al., 2016; Murakami et al., 2012).

ASC specks were remarkably reduced in the H‐VLN‐pretreated macrophages upon inflammasome activation (Figure 3c and Figure 3d). Therefore, H‐VLNs seemed to block ASC oligomerization and therefore prevented assembly of the NLRP3 inflammasome platform.

### 2.4 H‐VLNs protected mice from acute inflammatory conditions in the liver

**FIGURE 4:**
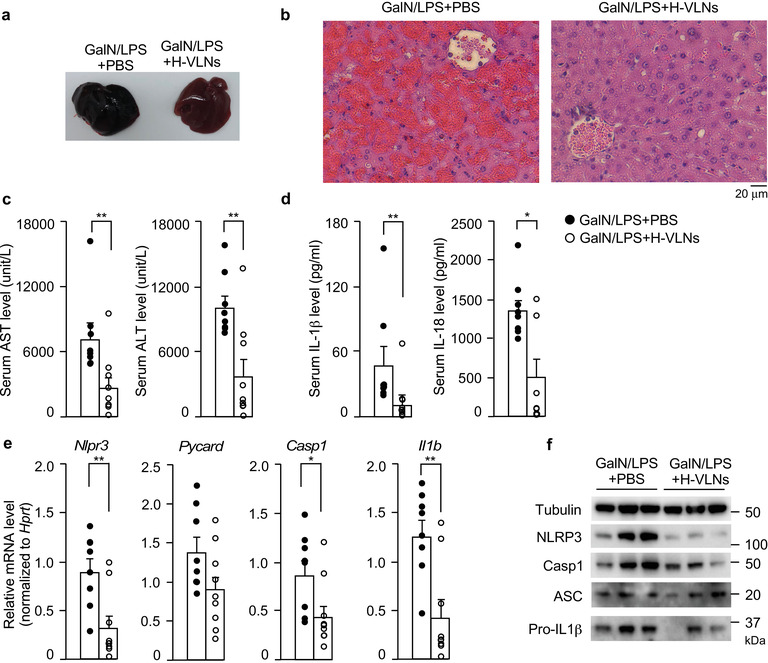
H‐VLNs alleviated inflammation and liver damage in GalN/LPS‐challenged mice. [8‐week‐old male C57BL/6J mice were intraperitoneally injected with solvent PBS or H‐VLNs at the dose of 0.3 × 10^10^/g in PBS. 48 h later, the mice were intraperitoneally injected with a mixture of GalN/LPS and sacrificed after 6 h for analysis. a. Representative image of whole mouse livers. b. Representative images of H&E staining of mouse livers. c. Serum levels of liver injury markers AST and ALT. d. Serum levels of cytokines IL‐1β and IL‐18. e. Expression of inflammatory genes in mouse livers. f. Immunoblot analysis of mouse livers. In the bar graphs, each dot represents one mouse. Data were presented as mean ± SEM. N = 8/group. * *P* < 0.05, ** *P* < 0.01 relative to mice challenged with GalN/LPS + PBS (bar with black dots)].

Because H‐VLNs showed potent anti‐NLRP3 inflammasome activity in cell culture, their anti‐inflammatory functions in vivo were assessed. Intraperitoneal injection of D‐galactosamine (GalN) and LPS triggers acute liver injury in mice, simulating many clinical characteristics of fulminant hepatic failure (Maes et al., 2016). Suppression of NLRP3 inflammasome activity improved the conditions of GalN/LPS‐challenged mice in other studies (Pourcet et al., 2018). Therefore, H‐VLNs were intraperitoneally injected in mice, followed by a GalN/LPS challenge. The livers from GalN/LPS‐treated mice were black (Figure 4a) due to excessive bleeding in the liver induced by the GalN/LPS challenge. Whole livers from GalN/LPS‐H‐VLN treated mice looked much more reddish and healthier compared with GalN/LPS‐challenged livers (Figure 4a). Haemotoxyline and Eosin (H&E)‐stained liver sections from GalN/LPS‐challenged mice showed dramatic pathological changes, including massive liver bleeding, hepatocyte damage, and cell shrinkage, which were largely curbed with H‐VLN pre‐treatment (Figure 4b). Circulating alanine aminotransferase (ALT) and aspartate aminotransferase (AST) are commonly used liver injury markers in clinical diagnosis and liver‐related research (McGill, 2016). Increases of both AST and ALT after the GalN/LPS challenge were alleviated by H‐VLNs (Figure 4c).

The plasma levels of both IL‐1β and IL‐18, two downstream products of NLRP3 inflammasome activation, were reduced after H‐VLN treatment (Figure 4d). H‐VLN treatment reduced the levels of IL‐6 and TNFα in mouse plasma, although IL‐6 reduction did not reach statistically significance (Figure S6a). H‐VLNs mitigated mRNA levels of the *Nlrp3, Casp1, Il1b, Il6, and Tnf* genes in the livers, but not levels of the *Pycard* (*Asc*) gene (Figure 4e, Figure S6b). Consistently, H‐VLNs decreased protein levels of NLRP3, Casp1, and pro‐IL‐1β in the livers, but had not much impact on ASC protein levels (Figure 4f).

### 2.5 RNAs in H‐VLNs were responsible for suppressing NLRP3 inflammasome activation

**FIGURE 5:**
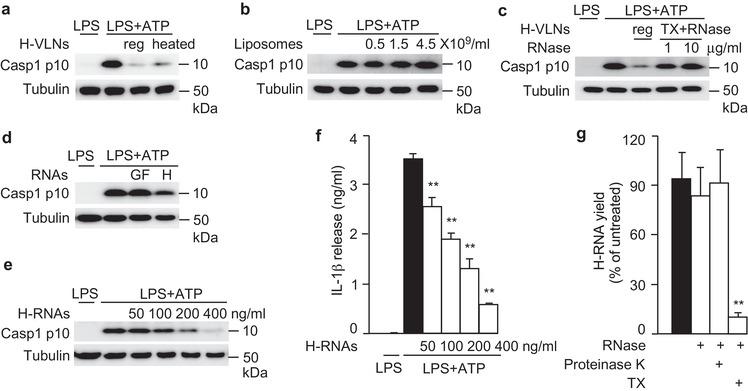
RNAs in H‐VLNs were responsible for suppressing NLRP3 inflammasome activity. [a. Proteins in H‐VLNs did not possess anti‐inflammasome function. H‐VLNs (1.5 × 10^9^/ml) were either untreated (reg) or heated at 95°C for 10 min (heated) to denature most proteins. b. Lipids from H‐VLNs were not required for anti‐inflammasome activity. Lipids were extracted from H‐VLNs and re‐assembled into liposomes. c. H‐VLNs depleted of RNAs lost anti‐inflammasome activity. H‐VLNs were either untreated (reg) or treated with Triton X‐100 (0.05%) and RNase (1 or 10 μg/ml) to deplete RNAs (TX + RNase). 1.5 × 10^9^/ml of H‐VLNs were used. d. RNAs from H‐VLNs, but not from GF‐VLNs, suppressed NLRP3 inflammasome activity. 400 ng/ml of RNAs were transfected in BMDMs. H: RNAs from H‐VLNs; GF: RNAs from GF‐VLNs. e‐f. RNAs from H‐VLNs (H‐RNAs) dose‐dependently inhibited Casp1 autocleavage (e) and IL‐1β secretion (f) upon inflammasome activation. 50‐400 ng/ml of RNAs from H‐VLNs were transfected in BMDMs, along with different amounts of RNAs from GF‐VLNs to ensure the total transfected RNAs of 400 ng/ml. After VLNs were added or RNAs were transfected for 16 h, BMDMs were treated with LPS + ATP to activate the NLRP3 inflammasome. g. Pretreatment with Triton X‐100 (TX) rendered RNAs in H‐VLNs sensitive to degradation by RNase. Intact H‐VLNs were either: untreated; treated with RNase alone; or pretreated with proteinase K (2 mg/ml) then heated to inactivate the enzyme, followed by RNase incubation; or pretreated with TX (0.1%) followed by RNase incubation. After all treatments, H‐VLNs were subjected to RNA extraction and RNA yields were measured. Data were presented as mean ± SEM. N = 3. * *P* < 0.05, ** *P* < 0.01 relative to macrophages treated with LPS + ATP (black bar) in f and ** *P* < 0.01 relative to H‐VLNs without any treatment (black bar) in g. Tubulin showed equivalent loading of cell lysates].

Different treatments were used to determine which category of biomolecules mediated the anti‐inflammasome function of H‐VLNs. First, H‐VLNs were heat treated to denature the proteins. The heat‐treated H‐VLNs reserved their ability to inhibit inflammasome‐mediated Casp1 autocleavage (Figure 5a), indicating that proteins in H‐VLNs did not mediate the anti‐inflammasome function. Total lipids were purified from H‐VLNs and re‐assembled into liposomes. Such liposomes did not blunt NLRP3 inflammasome activity (Figure 5b), suggesting that lipids in H‐VLNs were not responsible for suppressing inflammasome activity. RNAs in H‐VLNs were depleted using Triton X‐100 plus RNase treatment. Incubating membrane‐bound particles with Triton X‐100 compromised the membrane integrity, therefore making it easier for RNase to enter VLNs to degrade RNAs (Enderle et al., 2015; Ibrahim et al., 2014). Depletion of RNAs compromised the anti‐inflammasome potency of H‐VLNs (Figure 5c). To further confirm the importance of RNAs in H‐VLNs in inflammasome inhibition, total RNAs in H‐VLNs were extracted and transfected in BMDMs. RNAs from H‐VLNs inhibited inflammasome‐mediated Casp1 autocleavage, although RNAs from grapefruit‐derived VLNs (GF‐VLNs) did not influence inflammasome activity (Figure 5d). The intact GF‐VLNs and their RNAs had marginal impact on inflammasome activity (Figure S7a–S7c); therefore, their RNAs were used as a negative control. When different amounts of RNAs from H‐VLNs were transfected in BMDMs, they dose‐dependently inhibited both Casp1 autocleavage (Figure 5e) and IL‐1β release (Figure 5f) upon NLRP3 inflammasome activation. RNAs from H‐VLNs dramatically decreased secretion of IL‐6 and modestly reduced TNFα levels in the medium (Figure S7d and Figure S7e), but RNAs from GF‐VLNs mildly increased the secretion levels of IL‐6 and TNFα (Figure S7f and Figure S7g). Taken together, results of these experiments indicated that RNAs were the active biomolecules in H‐VLNs that inhibited NLRP3 inflammasome activity and NF‐κB signalling.

To verify that bioactive RNAs were present inside H‐VLNs, these particles were subjected to different treatments. RNase alone, which efficiently degraded the naked RNAs from H‐VLNs (Figure 1e), was not able to degrade RNAs in H‐VLN preparation (Figure 5g), suggesting that RNAs in H‐VLNs were well protected. Proteolytic digestion of the ribonucleoprotein complex could release RNAs from the protection of their binding proteins and therefore render them sensitive to RNase‐mediated degradation (Arroyo et al., 2011). However, after proteinase K digestion, RNAs in H‐VLN preparation still resisted degradation by RNase treatment (Figure 5g), indicating that RNAs were not stabilized through their protein‐binding partners. Proteinase K used in this study was verified to have strong proteolytic activity to effectively degrade proteins (Figure S8). When Triton X‐100 was used to compromise the membrane integrity, RNAs in H‐VLNs were substantially degraded by RNase (Figure 5g). Therefore, it seemed that RNAs were protected by the membrane‐bound structure of H‐VLNs.

### 2.6 miR‐4057 in H‐VLNs was identified in inhibiting NLRP3 inflammasome activity

RNAs in H‐VLNs were subjected to RNA deep sequencing. The raw sequencing data contained 6.9 M reads. After quality control and read filtering, 3,444,167 reads remained. Using miRDeep2 (Friedlander et al., 2012), the reads were first mapped to the entire miRBase sequence library (version 22) (Kozomara et al., 2019), which contains 48,885 known mature microRNAs (miRNAs) from 271 species and their precursors. According to miRDeep2's stringent mapping criteria (allowing 0 mismatch for precursor sequence mapping and ≤ 1 mismatch for mature sequence mapping using Bowtie), 4562 and 1047 reads were mapped to the hairpin and mature reference sequences, respectively, and 14 unique miRNAs were identified (Tier 1, Table S4). Among these miRNAs, the five most abundant miRNAs (Figure S9a and Table S4) were selected to synthesize their mimics. These mimics were transfected in BMDMs, followed by NLRP3 inflammasome activation. Among these miRNA candidates, miR‐1582 and miR‐5108 mildly inhibited NLRP3 inflammasome‐mediated Casp1 autocleavage (Figure S9b). Their respective inhibitors abolished the inhibitory function of miR‐1582 and miR‐5108 (Figure S9c and Figure S9d).

**FIGURE 6:**
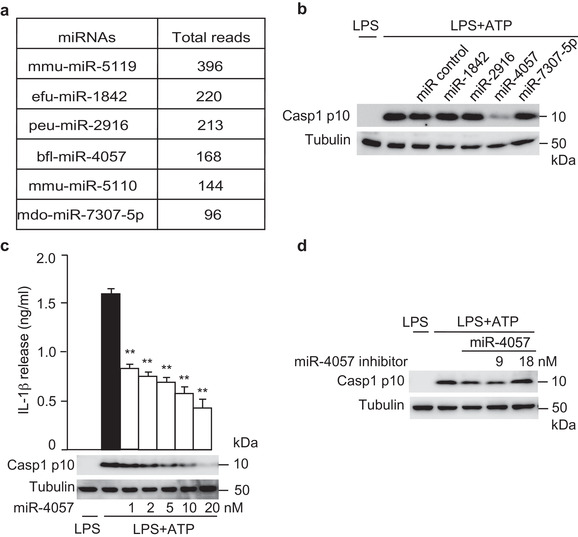
miR‐4057 in H‐VLNs was identified to suppress NLRP3 inflammasome activation. [a. Six most abundant miRNAs in H‐VLNs in Bowtie analysis. b. miR‐4057 inhibited inflammasome activation. 20 nM of miRNA mimic or miRNA (miR) negative control AllStars negative control siRNA were transfected. c. miR‐4057 dose‐dependently inhibited Casp1 autocleavage and IL1‐β secretion upon NLRP3 inflammasome activation. Different amounts of miRNA negative control were co‐transfected with miR‐4057 to ensure the total transfected RNAs of 20 nM. d. Inhibitor of miR‐4057 blunted the anti‐inflammasome activity of miR‐4057. 2 nM of miR‐4057 and different amounts of miRNA inhibitor and miR negative control were transfected to ensure the total RNAs of 20 nM. After 24 h, BMDMs were treated with LPS + ATP to activate the NLRP3 inflammasome. Data were presented as mean ± SEM. N = 3. * *P* < 0.05, ** *P* < 0.01 relative to macrophages treated with LPS+ATP (black bar). Tubulin showed equivalent loading of cell lysates].

The mild effects of miR‐1582 and miR‐5108 led us to run another bioinformatics analysis that mapped the reads against mature miRNA reference sequences alone using Bowtie (Langmead et al., 2009), which was less stringent compared to miRDeep2 and allowed more than one mismatch. Bowtie analysis identified 954 unique miRNAs (Tier 2, Table S5). The six most abundant miRNAs from Bowtie analysis were miR‐5119, miR‐1842, miR‐2916, miR‐4057, miR‐5110, and miR‐7307‐5p (Figure 6a). miR‐5119 showed no inhibition in the first round of test (Figure S9b). The RNA sequences from H‐VLNs matched nucleotides 7–25 of miR‐5110, which is out of the typical seed region (Bartel, 2009) and therefore was not further evaluated. Four other miRNA mimics were tested for their effects on the NLRP3 inflammasome. Interestingly, miR‐4057 potently suppressed Casp1 autocleavage during NLRP3 inflammasome activation (Figure 6b). Dose experiments showed that upon NLRP3 inflammasome activation miR‐4057 inhibited Casp1 autocleavage and IL‐1β release in a dose‐dependent manner (Figure 6c). However, miR‐4057 had no significant inhibition on the secretion of either IL‐6 or TNFα (Figure S10a–S10b). The inhibitor of miR‐4057 at a concentration of 18 nM abolished the inhibitory function of miR‐4057 (2 nM) (Figure 6d). We noted that RNA sequences in H‐VLNs mismatched the nucleotides 9–10 of the bfl‐miR‐4057 sequence in miRase. A miR‐4057 isoform that matched perfectly to RNA sequences in H‐VLNs was custom synthesized. This miR‐4057 isoform also dose‐dependently inhibited NLRP3 inflammasome‐mediated Casp1 autocleavage (Figure S10c), and its corresponding inhibitor blocked its inhibitory action on inflammasome activation (Figure S10d).

The source of bioactive miR‐4057 was further investigated. The manuka honey used in this study was obtained from bees who pollinate the native manuka bush in New Zealand. The total RNAs from H‐VLNs, honey bees, and manuka leaves were extracted and used to assess the miRNA levels. miR‐317‐3p and miR‐1‐3p, which are highly expressed in honey bees (Chen et al., 2010; Weaver et al., 2007), were included as positive miRNA controls for bee miRNAs. miR‐5119 and miR‐156a were used as positive honey miRNAs, because miR‐5119 was the most abundant miRNA in H‐VLNs and miR‐156a was detected in H‐VLNs and in other honeys in previous studies (Gismondi et al., 2017; Zhu et al., 2017). miR‐6478, reported as the most abundant miRNAs in GF‐VLNs (Xiao et al., 2018), was included as a negative control. Consistent with the high reads of miRNAs in deep sequencing data, miR5119, miR4057, and miR156a were readily detected in H‐VLN RNAs (Figure S10e). The levels of bee‐derived miR‐317‐3p and miR‐1‐3p were very low in H‐VLN RNAs (Figure S10e). In the bee RNAs, the miR‐1‐3p level was high, followed by miR‐317‐3p and miR‐156a, but the levels of other miRNAs were very low or undetectable (Figure S10f). In the manuka RNAs, miR4057 and miR156a were readily detected, but the levels of bee‐derived miR‐317‐3p and miR‐1‐3p were very low (Figure S10g). The level of miR‐6478 in all these RNA samples was extremely low or undetectable (Figure S10e–S10g). Therefore, miR‐4057 seemed to come from manuka trees.

## 3 DISCUSSION

In summary, we found that honey contained vesicle‐like nanoparticles, or VLNs. These nanoparticles from honey remarkably ameliorated NLRP3 inflammasome activity in primary macrophages. Administration of these nanoparticles in mice reduced inflammation and liver damage in experimentally induced acute liver injury. miR‐4057 in H‐VLNs was found to suppress the NLRP3 inflammasome.

Honey is mainly composed of sugars and water, although the exact composition of honey depends largely on its botanical origin (Bogdanov et al., 2008). Monosaccharides, including fructose and glucose, comprise approximately 95%–97% of the honey dry weight (Samarghandian et al., 2017). Besides sugars, honey contains minor components including minerals, vitamins, proteins, and polyphenols (Pasupuleti et al., 2017). Recently, miRNAs from honey bees (Zhu et al., 2017) and plants (Gismondi et al., 2017; Zhu et al., 2017) were found in honey. Here, we have identified a new bioactive component–VLNs–in honey. VLNs were found in six honeys from different sources; these VLNs were nanoparticles composed of proteins, lipids, and small‐sized RNAs (Figure 1 and Figure S1). Their size and composition were very similar to those of other dietary VLNs from edible plants and mushrooms (Chen et al., 2019; Liu et al., 2020; Mu et al., 2014; Zhang et al., 2016). The International Society for Extracellular Vesicles (ISEV) has recently defined EVs as ‘particles naturally released from the cell that are delimited by a lipid bilayer and cannot replicate’ (Thery et al., 2018). They should (1) contain at least one transmembrane or glycosylphosphatidylinositol‐anchored extracellular protein on the plasma membrane and/or endosome of eukaryotic cells; (2) include at least one cytosolic protein with the ability to bind to membrane lipids or to cytosolic sequences of transmembrane proteins; and 3) have low contamination of proteins from non‐EV structures co‐extracted with EVs (such as albumin as a common contaminant for serum EVs) (Thery et al., 2018). The demonstration of H‐VLNs as membrane‐bound particles under ultrastructure TEM and detergent lysis suggested the vesicle‐like nature of H‐VLNs. The presence of multiple plant‐originated plasma transmembrane proteins and membrane‐associated cytosolic proteins further suggested the potential identity of these particles in honey as *bona fide* vesicles. However, our proteomics analysis did not identify any tetraspanins. In mammalian cells, tetraspanins such as CD63 or CD81 are transmembrane proteins predominantly enriched in endosomes and used as well‐established EV markers for their released EVs (Yanez‐Mo et al., 2015). Failure to detect any plant‐derived tetraspanins in H‐VLNs could be attributed to the difficulty of recovering peptides from this type of highly hydrophobic proteins and limitations of the proteomics approach used or to the intrinsic absence of these proteins in H‐VLNs. However, unavailability of commercial antibodies against any plant‐derived tetraspanins and lack of a reference proteome database for the manuka tree have hindered further validation of the presence of any CD63 or CD81 plant homologs in H‐VLNs. Similar to other dietary VLNs (Teng et al., 2018; Xiao et al., 2018), H‐VLNs contain a series of miRNAs. miR‐156a, a miRNA identified in H‐VLNs (Figure S9a and Table S4), was also found in honeys from different sources by two other research groups (Gismondi et al., 2017; Zhu et al., 2017). It is tempting to speculate that miRNAs in honey are likely packaged in VLNs since encapsulation of miRNAs in VLNs confers protection against degradation.

Honey has been shown to have anti‐microbial, anti‐oxidant, and anti‐inflammatory bioactivities (Bogdanov et al., 2008; Samarghandian et al., 2017). As evidence of its anti‐inflammatory activities, honey has been found to reduce inflammation and neutrophil infiltration in the colon in trinitrobenzene sulfonic acid (TNBS)‐induced colitis in rats (Bilsel et al., 2002; Prakash et al., 2008). The phenolic extract of honey inhibits expression of the *Tnf* and *Il1b* genes induced by LPS in N13 microglial cells (Candiracci et al., 2012). Certain types of indigenous honey from New Zealand decrease the superoxide production of human neutrophils and alleviate arachidonic acid‐induced ear edema and immune cell infiltration in mice (Leong et al., 2012). In HFD‐challenged rats, honey ameliorates chronic hepatic injury and reduces inflammation in the liver (Xiao et al., 2016). At the molecular level, honey has been shown to decrease the release of IL‐1β and IL‐18 (downstream products of the NLRP3 inflammasome) in the livers of HFD‐fed rats and in the free fatty acid‐treated rat hepatocyte BRL‐3A cell line (Xiao et al., 2016), indicating possible regulatory effects of honey on the NLRP3 inflammasome. However, the direct effects of honey on the NLRP3 inflammasome had not been demonstrated. In our studies, we found that VLNs from honey specifically inhibited activity of the NLRP3 inflammasome (Figure 2) by preventing formation of the inflammasome complex (Figure 3). Therefore, our research has clearly defined the role of H‐VLNs in suppressing activity of the NLRP3 inflammasome and has elucidated that honey inhibits inflammation through a new target – the NLRP3 inflammasome. It is plausible that the anti‐inflammatory bioactivities of honey identified in previous studies are mediated at least partially through inhibition of NLRP3 inflammasome activity.

miRNAs in dietary VLNs have been found to play various roles. miRNA‐7267‐3p in ginger‐derived VLNs has been found to suppress expression of the *ycnE* gene in gut microbiota *Lactobacillaceae*, leading to a change of tryptophan metabolism in these bacteria (Teng et al., 2018). miR‐167a, another miRNA in ginger‐derived VLNs, inhibited transcription of a Pili gene, *SpaC* in *Lactobacillaceae* and therefore reduced bacterial mobilization from the gut to other organs (Teng et al., 2018). miRNAs from ginger‐derived VLNs, including miR‐159a, miR‐166u, and miR‐166p, have been shown to inhibit proliferation of a periodontal pathogen, *Porphyromonas gingivalis* (Sundaram et al., 2019). Ultrasonication of bovine skim milk leads to a >98% depletion of miRNAs in the milk EVs and a 20% depletion of milk EVs (Leiferman et al., 2018; Wu et al., 2019). When such ultrasonicated skim milk was supplemented in a mouse diet, the expressions of approximately 10 genes in skeletal muscle were altered, compared to mice fed with a diet supplemented with untreated skim milk (Leiferman et al., 2018). The same dietary depletion of milk EVs and their miRNA cargos elevated intestinal inflammation and altered transcription of 16 immune genes in *Mdr1a* ^–/–^ mice (Wu et al., 2019). However, it is not clear whether depletion of miRNAs in the milk EVs influences directly transcription in consumers or through other mechanisms (such as reshaping the microbiota in the gut). Our studies demonstrated that RNAs in H‐VLNs are necessary and sufficient for suppressing NLRP3 inflammasome activity (Figure 5). Further screening of miRNAs revealed that miR‐4057 in H‐VLNs suppressed NLRP3 inflammasome activation in macrophages (Figure 6). Therefore, our research has demonstrated that miRNAs from another dietary source, H‐VLNs, possess intrinsic anti‐inflammatory activities.

The role of dietary miRNAs in consumers is highly controversial. An early report suggested that rice‐originated miR‐168a was enriched in mouse serum and liver after rice consumption, accompanied by decreased levels of low‐density lipoprotein receptor adaptor protein 1 (LDLRAP1) in the liver samples (Zhang et al., 2011). However, because of conflicting follow‐up data in this area, the concept of dietary transfer of miRNAs has been the subject of intense debate (Witwer & Hirschi, 2014; Witwer & Zhang, 2017). Although miRNAs in dietary VLNs seem to regulate the functions and/or population of microbiota in the gut of consumers (Teng et al., 2018; Zhou et al., 2019), corroborating and proverbial evidence is needed to support their direct regulatory role in consumers. In our studies, intraperitoneal injection of H‐VLNs alleviated inflammation in experimentally induced acute liver injury in mice (Figure 4), providing proof‐of‐principle demonstration of the anti‐inflammatory functions of H‐VLNs in vivo. However, further studies are warranted to investigate whether oral administration of H‐VLNs and their bioactive component miRNAs could be absorbed through the gastrointestinal tract and exert any anti‐inflammatory function in consumers.

Despite the well‐documented nutraceutical values of honey, some adverse effects of honey have been reported due to contamination from pesticides, antibiotics, and heavy metals introduced through disease control, environmental exposure, and processing (Ajibola et al., 2012; Bogdanov et al., 2008). The high sugar content of honey poses the theoretical risk of increasing blood glucose levels in diabetic patients. Indeed, in clinical trials, consumption of honey for 8 weeks increased the level of blood haemoglobin A1c (HbA1c) in patients with type 2 diabetes (Bahrami et al., 2009; Sadeghi et al., 2019), suggesting that extra caution is needed when honey is consumed by diabetic patients. The H‐VLN extraction process removes sugars and likely most of the contaminated agrochemicals in honey, although H‐VLNs retain their potent anti‐inflammatory bioactivities and possibly other functions. Therefore, H‐VLNs could be potentially used to achieve at least some of the medicinal functions of honey in vulnerable populations such as diabetic patients.

In conclusion, VLNs with strong anti‐inflammatory functions represent a new type of bioactive agent identified in honey. This finding opens a new avenue for studying honey, a medicinal food since ancient time. Many questions await further investigation. Besides the studies raised earlier, further studies are necessary to determine the molecular mechanisms by which H‐VLNs prevent inflammasome formation and activation. It would be also important and interesting to investigate other potential functions of H‐VLNs and their possible interactions with microbiota in the gut.

## 4 MATERIALS AND METHODS

### 4.1 Honey bees, honey, and other hive products

Twenty colonies of European honey bees (*Apis mellifera L*.) were maintained with standard Langstroth equipment at the University of Nebraska‐Lincoln East Campus Pollinator Garden (Lincoln NE, USA). Beehive samples, including fresh unprocessed honey, royal jelly, and freshly collected forage (nectar and pollen), were harvested from 3 to 4 randomly selected queenright and healthy colonies and immediately subjected to VLN extraction. Five commercially available honeys, including manuka honey (unprocessed, product of New Zealand, distributed by Wedderspoon, Malvern, PA, USA); two local Nebraska honeys (unprocessed or lightly heated and filtered, product of Nebraska USA, distributed by It's all about bees!, Ralston, NE, USA); two organic honeys (unfiltered or filtered, product of Brazil and Mexico, distributed by Wholesome Sweeteners, Sugar Land, TX, USA) were purchased from the local Whole Foods Market (Lincoln, NE, USA).

### 4.2 Isolation of VLNs

Honeys, other hive products, and grapefruit were subjected to VLN extraction as described with minor modifications (Chen et al., 2019; Mu et al., 2014; Wang et al., 2018). A total of 2 g of pollen or grapefruit pulps were ground for 15 sec in cold PBS in a kitchen blender, while 2 g of honey, nectar, or royal jelly were diluted with cold PBS. The diluent underwent sequential centrifugation at 500 × *g* for 10 min, 2000 × *g* for 20 min and 10,000 × *g* for 30 min. The supernatant was subjected to ultracentrifugation at 100,000 × *g* for 2 h. The nanoparticle pellets were washed with cold PBS, resuspended in PBS or medium, and filtered through a 200 nm Acrodisc filter (Pall Laboratory, Port Washington, NY, USA). Alternatively, VLNs in PBS were loaded on the top of a sucrose gradient (8%: 30%: 60% from the top to bottom) and underwent ultracentrifugation at 150,000 × *g* for 16 h. Fractions were carefully collected at 1 ml each (Fractions 1–11 from top to the bottom). The particle number in each fraction was measured. The pellet at the bottom of the tube was washed twice with PBS and resuspended in PBS for further analysis. The fractionated particles were diluted with PBS and ultracentrifuged at 100,000 × *g* for 2 h to collect the nanoparticles.

### 4.3 Characterization of VLNs

A NanoSight NS300 instrument (Malvern, Westborough, MA, USA) was used to measure size and yield of VLNs as described (Chen et al., 2019). RNAs were extracted from VLNs using miRNeasy Mini kit (Qiagen, Germantown, MD, USA) and run on a 2.5% agarose gel. Proteins were extracted from VLNs using lysis buffer containing 150 mM NaCl, 0.5% NP‐40, 50 mM Tris‐HCl (pH7.5), resolved on a Bis‐Tris protein gel (Invitrogen, Carlsbad, CA, USA), and visualized with Coomassie blue staining. Lipids were purified from VLNs using the Folch method (Folch et al., 1957; Zhuang et al., 2015), run on a silica gel TLC plate (EMD Millipore, Burlington, MA, USA) using a solvent mixture of chloroform /methanol /acetic acid (190:9:1, Sigma, St. Louis, MO, USA), and stained with 10% CuSO_4_ in 8% phosphoric acid (Sigma). Total lipids of H‐VLNs were used to prepare liposomes as described (Chen et al., 2019).

For the Triton X‐100 lysis experiment, H‐VLNs in PBS were aliquoted into 5 tubes, to which were added 0, 0.01%, 0.025%, 0.05% or 0.1% (v/v in PBS) of Triton X‐100, followed by vigorous agitation using a vortex mixer at highest speed for 30 sec. The mixture was incubated at room temperature for 30 min. The samples were vortexed again at highest speed for 30 sec and subject to particle number measurement.

To determine if RNAs were present inside H‐VLNs, H‐VLNs in PBS were aliquoted into 4 tubes. One sample was not subjected to any treatment and served as control. One sample was incubated with RNase (10 μg/ml) for 30 min at 37 °C. One sample was incubated with proteinase K (2 mg/ml) for 30 min at 37°C, followed by heat treatment at 95 °C for 5 min to denature proteinase K, and another 30 min incubation with RNase (10 μg/ml) at 37°C. The last sample was added to 0.1% (v/v) of Triton X‐100, vortexed vigorously, and incubated at room temperature for 30 min, followed by 30 min incubation with RNase (10 μg/ml) at 37°C. After all treatments, the samples were subjected to RNA extraction using miRNeasy Mini kit, and RNA concentration was measured.

### 4.4 Electron microscopy analysis of VLNs

H‐VLNs purified from the sucrose gradient fractionation were subjected to electron microscopy analysis. SEM analysis of H‐VLNs was conducted as previously described (Chen et al., 2019). SEM images of H‐VLNs were obtained using a Hitachi S4700 Field‐Emission SEM (Hitachi, Santa Clara, CA, USA). For ultrastructure TEM analysis, H‐VLN particle pellets were incubated with 2.5% glutaraldehyde in 0.1 M cacodylate buffer (pH 7.2) for 1 h at room temperature and embedded with 2% low‐melting agarose gel to minimize the loss of particles during a series of processing steps. The lipids in the VLNs were post‐fixed using 1% osmium tetroxide at room temperature for 1 h, then washed three times with deionized water. A graduated ethanol series was used to dehydrate the samples, which were eventually embedded in Spurr medium mix (Electron Microscopic Sciences, Fort Washington, PA, USA), and cut into 90 nm‐thick sections using a Leica UC7 ultramicrotome (Allendale, NJ, USA). The sections were stained with uranyl acetate and lead citrate and checked using Hitachi H7500 TEM with a bottom‐mount AMT digital camera.

### 4.5 H‐VLN RNA library preparation and deep sequencing

RNAs extracted from H‐VLNs with a miRNeasy Mini kit (Qiagen) were used to generate a small RNA library with a NEXTflex Small RNA‐seq Kit V3 (BiooScientific, Austin, TX, USA) according to the manufacturer's instructions. Briefly, RNAs were desalted and treated with T4 polynucleotide kinase, and 12 ng of processed RNAs were subjected to 3’ adapter ligation overnight at 20 °C. Following 20‐cycle PCR amplification of the library, the sample was analyzed using the Fragment Analyzer and quantitated using qPCR. A total of 8 pM of pooled library were sequenced on a MiSeq system (Illumina, San Diego, CA, USA) using the MiSeq reagent Kit V3 (Illumina) for 150 cycles. The sequencing data obtained were first assessed for the read quality control using FastQC (FastQC, 2010). Cutadapt (Martin, 2011) was used to remove adapter sequences and bases with quality scores of less than 10. Only reads with lengths between 18–40 base pairs were kept as quality reads, which were first mapped using miRDeep2 (Friedlander et al., 2012) to the known 38,589 hairpin sequences and annotated according to 48,885 known mature miRNAs from 271 species (miRBase database (Kozomara et al., 2019) version 22). A less stringent mapping against all known mature reference with more than one mismatch allowed was performed using Bowtie (Langmead et al., 2009).

### 4.6 H‐VLN protein digestion and proteomics analysis

H‐VLN pellets were resuspended in NuPAGE LDS sample buffer (ThermoFisher Scientific, Waltham, MA), heated at 95°C for 5 min, alkylated with 15 mM iodoacetamide for 15 min at room temperature, and run in a Bolt 12% Bis‐Tris Plus gel for 10 min to concentrate the proteins and separate any contaminants. The proteins were in‐gel digested with trypsin for 16 h at 37°C, and resulting peptides were analyzed with LC‐MS/MS using a RSLCnano system (ThermoFisher Scientific) coupled to a Q‐Exactive HF mass spectrometer (ThermoFisher Scientific). Proteomics data were analyzed with Mascot v 2.6.1 (Matrix Science, Boston, MA, USA) and searched using the common contaminants database cRAP (123 entries, www.theGPM.org), the Uniprot reference proteome database for *Apis mellifera* (retrieved on 04/30/2019, 15321 entries), and the Uniprot entries for *viridiplantae* (retrieved on 04/30/2019, 7108705 entries). Scaffold v4.8.9 (Proteome Software Inc., Portland, OR) was used to validate LC‐MS/MS‐based peptide and protein identifications. Peptide identifications were accepted if they could be established at greater than 80.0% probability by the Peptide Prophet algorithm (Keller et al., 2002) with Scaffold delta‐mass correction. Protein identifications were accepted if they could be established at greater than 99.0% probability by the ProteinProphet algorithm (Nesvizhskii et al., 2003) and contained at least two identified peptides.

### 4.7 Macrophage culture

The primary macrophage BMDMs from C57BL/6J mice were prepared as described (Yu et al., 2014). To study the role of dietary VLNs in regulating NLRP3 inflammasome activity, BMDMs were treated with VLNs for 16 h, then incubated with the priming signal LPS (InvivoGen, San Diego, CA, USA, tlrl‐peklps, 10 ng/ml) for 3 h followed by treatment with activating stimulus. ATP (Sigma, 5 mM, 30 min), nigericin (Enzo Life Sci, Farmingdale, NY

USA, 5 μM, 30 min), free fatty acid sodium palmitate (Sigma, 1mM, 16 h), and alum (ThermoFisher Scientific, 0.5% v/v, 5 h) were used to activate the NLRP3 inflammasome. BMDMs were primed with LPS for 3 h, then transfected with calf DNA (Sigma, D3664, 2 μg/well) for 2 h to induce activation of the AIM2 inflammasome. After inflammasome activation, the cell culture medium was collected and centrifuged at 300 × *g* for 5 min at 4°C to remove any cell debris. The supernatant was used for cytokine measurement. The cells were directly lysed in SDS loading buffer, heated at 95°C for 10 min, and subjected to immunoblot analysis.

### 4.8 Mice

C57BL/6J mice were purchased from Jackson Laboratory (Bar Harbor, ME, USA) and were grown in an animal facility free of specific pathogens. Animal experiments were performed in accordance with the protocol (Project ID 1421) approved by the Institutional Animal Care and Use Committee of University of Nebraska‐Lincoln. To assess the anti‐inflammatory function of H‐VLNs in mice, 8‐week‐old male C57BL/6J mice were intraperitoneally injected with H‐VLNs at a dose of 0.3 × 10^10^/g. 48 h later, mice were intraperitoneally administered a mixture of D‐galactosamine (GalN, Sigma, 34539, 500 mg/kg) and LPS (Sigma, L2630, 15 μg/kg) to trigger acute liver injury; 6 h after GalN/LPS injection, all mice were sacrificed, and plasma and liver tissues were taken for further analysis. The levels of ALT and AST in circulation were assessed using a Vitros‐250 Chemistry Analyzer (Johnson&Johnson, New Brunswick, NJ, USA).

### 4.9 ASC speck staining and ASC oligomerization assay

ASC speck staining and ASC oligomerization assay were carried out as described (Chen et al., 2019). The primary anti‐ASC antibody (Adipogen, San Diego, CA, USA, AG25B0006C100, 1:200) and Alexa‐Fluor‐594‐conjugated secondary antibody (Invitrogen, A‐11037, 1:200) were used. Cell images were obtained using an A1R‐Ti2 confocal system (Nikon).

### 4.10 Immunoblot analysis

Protein lysates from cells or liver tissues were separated on a 4%–12% NuPAGE Bis‐Tris protein gel (Invitrogen) and transferred to a polyvinylidene difluoride membrane (GE Healthcare, Chicago, IL, USA). Blots were blocked with 5% nonfat milk (Nestle, Jacksonville, IL, USA), then incubated with primary antibodies in Tris‐buffered saline containing 0.1% Tween 20 (Sigma) and 5% nonfat milk, followed by HRP‐conjugated anti‐rabbit antibody (Cell Signaling, Danvers, MA, USA, 7074S, at 1:3000 dilution) or anti‐mouse antibodies (Cell Signaling, 7076S, at 1:2500 dilution). Primary antibodies used in this study were anti‐NLRP3 mouse antibody (Adipogen, AG20B0014C100, 1:1000); anti‐ASC rabbit antibody (Adipogen, AG25B0006C100, 1:1000); anti‐Casp1 (p10) mouse antibody (Adipogen, AG20B0044C100, 1:1000); anti‐tubulin rabbit polyantibody (Santa Cruz, Dallas, TX, USA, SC‐5286, 1:200); anti‐Nek7 rabbit antibody (Abcam, Cambridge, MA, USA, ab133514, 1:10000); and anti‐IL‐1β goat antibody (R&D systems, Minneapolis, MN, USA, AF401NA, 1:2000).

### 4.11 RNA extraction and quantitative PCR (qPCR)

For RNA extraction, liver tissues of mice were snap frozen in liquid nitrogen upon sacrifice and kept at ‐80°C. The live worker bees were collected and sacrificed. Their guts and eyes were removed to avoid contamination from microbiota in the gut and pigments in the eyes. The rest of the bee bodies were snap frozen in liquid nitrogen and ground into fine powder using a pestle and a mortar soaked in liquid nitrogen. Manuka tree leaves (*Leptospermum scoparium*) were rinsed in deionized water three times and once in 70% ethanol, then frozen in liquid nitrogen. The leaves were finely ground into powder using a pestle and a mortar soaked in liquid nitrogen. RNA‐bee (Tel‐Test, Friendswood, TX, USA) was used to extract total RNAs from mouse liver and bees per manufacturer's protocol. The powder of manuka tree leaves was homogenized in TRIzol Reagent (Ambion, Austin, TX, USA) and incubated for 5 min at room temperature. 1/5 volume of chloroform was added, followed by vigorous agitation for 15 sec and incubation for 3 min. The samples were centrifuged at 12,000 × *g* for 15 min at 4°C, and the upper aqueous phase was collected and mixed with 1/10 volume of sodium acetate (pH5.2) and equal volume of isopropyl alcohol. The samples were kept at ‐20°C for 16 h and centrifuged at 15,000 × *g* for 20 min at 4°C to collect RNAs, which were washed with 75% ethanol twice and dried. The obtained RNAs were treated with DNase I to remove possible genomic DNA contamination and further purified using PureLink RNA Mini Kit (Ambion) according to the manufacturer's protocol.

For mRNA analysis, cDNA synthesis was conducted using a high‐capacity cDNA Reverse Transcription Kit (Applied Biosystems, Foster City, CA, USA), and qPCR was done using a CFX Connect Real‐time System (Bio‐Rad, Hercules, CA, USA). Relative mRNA levels were assessed by normalizing the gene of interest to the hypoxanthine guanine phosphoribosyl transferase (*Hprt*) gene. For miRNA analysis, a Spike‐in control (Qiagen, 219610) was used as an internal control to calibrate amplification efficiency. A specific reverse transcript primer that detected individual miRNA was designed based on the stem‐loop method (Cheng et al., 2016; Kramer, 2011) (Table S6). A total of 250 ng of total RNAs were used as a template for each reverse transcription reaction. qPCR was performed using the primers shown in Table S6, and relative miRNA levels were expressed by normalizing the miRNA of interest to the Spike‐in control.

### 4.12 miRNA transfection

Total RNA from H‐VLNs or GF‐VLNs or miRNA mimics were transfected in BMDMs using Lipofectamine RNAiMAX (Invitrogen) per manufacturer's protocol. Briefly, 2 μl of Lipofectamine RNAiMAX were diluted in Opti‐MEM (ThermoFisher Scientific) and added to 20 nM of miRNA mimic (Qiagen) or AllStars negative control siRNA (Qiagen) in Opti‐MEM. After 20 min of incubation, the mixture was added to the BMDMs in a 24‐well plate. After 24 h of transfection, the cells were treated with LPS + ATP to activate the NLRP3 inflammasome. 400 ng/ml of total RNAs from H‐VLNs or GF‐VLNs were transfected in BMDMs using 2 μl of Lipofectamine RNAiMAX. For the dose experiment, different amounts of GF‐VLN RNAs were added to H‐VLN RNAs to ensure the total transfected RNAs at the concentration of 400 ng/ml. Alternatively, different amounts of negative control miRNA were added to miR‐4057 to ensure the total transfected RNAs at the concentration of 20 nM.

### 4.13 Enzyme‐linked immunosorbent assay (ELISA) and LDH release

ELISA analysis and LDH release were carried out as described (Chen et al., 2019). The ELISA kits used included IL‐1β (eBioscience, San Diego, CA, USA, 88701388); IL‐18 (MBL, Worburn, MA, USA, D042‐3); IL‐6 (BioLegend, San Diego, CA, USA, 431301); and TNFα (BioLegend, 430901). LDH release was measured using a CytoTox 96 Nonradioactive Cytotoxicity Assay kit (Promega, Madison, WI, USA).

## 5 STATISTICS

Data for the cell culture experiments were analyzed using Excel software. Differences between the two groups were compared using a two‐tailed t test. *P* < 0.05 was indicated by * and considered significant. *P* < 0.01 was indicated by **. All cell culture experiments were repeated at least 3 times. The animal experiments were analyzed using R version 3.6.0 (R Core Team, The R Foundation for Statistical Computing, Vienna, Austria) (Liu et al., 2020). The Shapiro‐Wilks test was used to evaluate if the data were normally distributed. As the Shapiro‐Wilks test showed that the data were not normally distributed, differences between the two groups were compared using the nonparametric Mann‐Whitney test. *P* < 0.05 was indicated by * and considered significant. *P* < 0.01 was indicated by **.

## CONFLICTS OF INTEREST

The authors report no conflict of interest.

## Supporting information



Supporting Information

Supporting Information

Supporting Information

Supporting Information

Supporting Information

Supporting Information
