# Supplementary material for: Identification of anti‐inflammatory vesicle‐like nanoparticles in honey
Source: J Extracell Vesicles. 2021 Feb 12;10(4):e12069. doi: 10.1002/jev2.12069 (PMC7879699; doi:10.1002/jev2.12069)
Supplement: Supplementary file 1 — Supporting Information [file JEV2-10-e12069-s001.docx]

|  |
| --- |
| **Supplementary Figure 1. Honeys from different sources contained VLNs. a**. Sizes of VLNs from five honeys, including UNL-fresh, NE-un, NE-processed, Mixed-un, and Mixed-processed. **b**-**d**. VLNs from UNL-fresh, NE-un, and NE-processed were used to extract RNAs, proteins, and lipids and these biomolecules were separated and visualized on RNA gel (**b**), protein gel (**c**), and TLC plate (**d**). un: unprocessed. |

|  |
| --- |
| **Supplementary Figure 2. H-VLNs from manuka honey inhibited IL-6 secretion and expression of inflammatory genes. a**-**b.** BMDMs were pretreated with H-VLNs for 16 h, then LPS+ATP was added to activate the NLRP3 inflammasome. H-VLNs inhibited IL-6 secretion (**a**), but not TNFα secretion (**b**). **c-e.** BMDMs were pretreated with H-VLNs for 16 h, followed by LPS incubation for 3 h. Cells were collected for mRNA extraction and qPCR analysis. H-VLNs inhibited expression of the *Il6, Tnf*, and *Il1b* genes. Data were presented as mean±SEM. N=3. *p<0.05, **p<0.01 relative to BMDMs treated with LPS+ATP (black bar) in **a**-**b** and relative to BMDMs treated with LPS (black bar) in **c**-**e**. un: BMDMs without any treatment. |

|  |
| --- |
| **Supplementary Figure 3. VLNs from most honeys suppressed NLRP3 inflammasome activation. a-b.** VLNs from fresh local honey UNL-fresh inhibited IL-1β release (**a**) and Casp1 autocleavage (**b**) upon inflammasome activation. **c**. VLNs from some commercial honeys blunted IL-1β secretion. **d**. VLNs from local honey NE-un inhibited Casp1 autocleavage. **e**. VLNs from local honey NE-processed inhibited Casp1 autocleavage. **f**. VLNs from honey Mixed-un had marginal effects on Casp1 autocleavage. **g**. VLNs from honey Mixed-processed inhibited Casp1 autocleavage. BMDMs were incubated with H-VLNs from different sources for 16 h, followed by NLRP3 inflammasome activation with LPS+ATP. Tubulin showed equivalent loading of cell lysates. Data were presented as mean±SEM. N=3. *p<0.05, **p<0.01 relative to BMDMs treated with LPS+ATP (black bar). un: unprocessed. |

|  |
| --- |
| ****  **Supplementary Figure 4. VLNs from five honeys increased IL-6 secretion.** VLNs from five honeys increased IL-6 release (**a, c**), but had no impact on TNFα secretion (**b, d**) upon NLRP3 inflammasome activation. BMDMs were incubated with H-VLNs from different sources for 16 h, followed by NLRP3 inflammasome activation using LPS+ATP. Data were presented as mean±SEM. N=3. *p<0.05, **p<0.01 relative to BMDMs treated with LPS+ATP (black bar). un: unprocessed. |

|  |
| --- |
| **Supplementary Figure 5. Effects of other beehive products on activation of the NLRP3 inflammasome. a**. VLNs extracted from freshly collected nectar inhibited NLRP3 inflammasome-mediated Casp1 autocleavage. **b**. VLNs extracted from freshly collected pollen had no impact on Casp1 autocleavage upon inflammasome activation. **c**. VLNs extracted from royal jelly had marginal effects on Casp1 autocleavage. BMDMs were incubated with VLNs from different hive products for 16 h, followed by NLRP3 inflammasome activation with LPS+ATP. Tubulin showed equivalent loading of cell lysates. |

|  |
| --- |
| ****  **Supplementary Figure 6. H-VLNs reduced TNFα level in serum and suppressed expression of the *Il6* and *Tnf* genes in livers in GalN/LPS-challenged mice.** The serum and livers of the mice in Fig. 4 were further analyzed. **a**. Serum levels of cytokines IL-6 and TNFα. **b**. Expression of the *Il6* and *Tnf* genes in mouse livers. In the bar graphs, each dot represents one mouse. Data were presented as mean±SEM. N = 8/group. * p <0.05, ** p <0.01 relative to mice challenged with GalN/LPS+PBS (bar with black dots). |
|  |
| ****  **Supplementary Figure 7. Effects of RNAs from GF-VLNs on NLRP3 inflammasome activation and cytokine secretion. a.** Intact GF-VLNs had no impact on NLRP3 inflammasome-mediated Casp1 autocleavage. **b-c**. RNAs from GF-VLNs had no impact on Casp1 autocleavage (**b**) and IL-1β secretion (**c**) upon NLRP3 inflammasome activation. **d-e**. RNAs from H-VLNs inhibited the levels of IL-6 (**d**) and TNFα (**e**). **f-g**. RNAs from GF-VLNs increased the levels of IL-6 (**f**) and TNFα (**g**). BMDMs were incubated with GF-VLNs or transfected with RNAs from VLNs for 16 h, followed by NLRP3 inflammasome activation with LPS+ATP. Data were presented as mean±SEM. N=3. *p<0.05, **p<0.01 relative to BMDMs treated with LPS+ATP (black bar). Tubulin showed equivalent loading of cell lysates. |

|  |
| --- |
| **Supplementary Figure 8. Proteolytic activity of proteinase K.** Proteinase K was untreated (active) or heated at 95 °C for 5 min (heat-treated). Different amounts of active or heated proteinase K were incubated with 200 μg of bovine serum albumin (BSA) for 30 min at 37 °C. The samples were run on a 4-12% Bis-Tris protein gel and visualized with Coomassie blue. |

|  |
| --- |
| **Supplementary Figure 9.** **miR-1582 and miR-5108 in H-VLNs mildly suppressed NLRP3 inflammasome activation. a.** Five most abundant miRNAs in H-VLNs in miRDeep2 analysis. **b**. miR-1582 and miR-5108 mildly inhibited inflammasome activation. 20 nM of miRNA mimic or miRNA (miR) negative control AllStars negative control siRNA were transfected. **c**. Inhibitor of miR-1582 abolished the ability of miR-1582 to inhibit the NLRP3 inflammasome. **d**. Inhibitor of miR-5108 blunted the anti-inflammasome activity of miR-5108. In the inhibitor experiments, 5 nM of miR-1582 or miR-5108 and different amounts of miRNA inhibitor and miR negative control were used to ensure the total transfected RNA of 20 nM. After 16 h, BMDMs were treated with LPS+ATP to activate the NLRP3 inflammasome. Tubulin showed equivalent loading of cell lysates. |

|  |
| --- |
| **Supplementary Figure 10.** **Action of miR-4057 and miR-4057 isoform and origination of miR-4057 isoform. a-b**. miR-4057 increased the levels of IL-6 (**a**) and had no impact on TNFα (**b**). **c**. miR-4057 isoform (iso) dose-dependently inhibited NLRP3 inflammasome-mediated Casp1 autocleavage. Different amounts of miRNA negative control were co-transfected with miR-4057 isoform to ensure the total transfected RNAs of 20 nM. **d**. Inhibitor of miR-4057 isoform blunted the anti-inflammasome activity of miR-4057 isoform. 2 nM of miR-4057 isoform and different amounts of miRNA inhibitor and miR negative control were used to ensure the total transfected RNAs of 20 nM. After 24 h, BMDMs were treated with LPS+ATP to activate the NLRP3 inflammasome. **e**-**g**. Relative levels of miRNAs in RNAs extracted from H-VLNs (**e**), worker bees (**f**), and manuka tree leaves (**g**). Relative levels of miRNAs were expressed by normalizing miRNA of interest to the Spike-in control. Data were presented as mean±SEM. N = 3. * p<0.05, ** p<0.01 relative to macrophages treated with LPS+ATP (black bar). |
