# Supplementary material for: Identification of anti‐inflammatory vesicle‐like nanoparticles in honey
Source: J Extracell Vesicles. 2021 Feb 12;10(4):e12069. doi: 10.1002/jev2.12069 (PMC7879699; doi:10.1002/jev2.12069)
Supplement: Supplementary file 2 — Supporting Information [file JEV2-10-e12069-s002.docx]

**Supplementary Table 1. Yield of VLNs in six honeys.**

| **Honeys** | Manuka | UNL-  fresh | NE-unprocessed | NE-processed | Mixed-unprocessed | Mixed-  processed |
| --- | --- | --- | --- | --- | --- | --- |
| **VLN yield (×10^10^/g)** | 7.5±1.6 | 0.9±0.1 | 4.1 ±1.3 | 3.7±0.3 | 8.5±0.6 | 5.1±0.3 |

**Supplementary Table 2. Concentrations of biomolecules in H-VLNs.**

| **Biomolecules** | RNAs (ng/10^10^) | Proteins (μg/10^10^) | Lipids (μg/10^10^) |
| --- | --- | --- | --- |
| **yield** | 30.7±1.1 | 9.2±1.0 | 216.4 ±6.3 |
